# Interleukin-22 acts as a mitochondrial protector

**DOI:** 10.7150/thno.48022

**Published:** 2020-06-19

**Authors:** Seonghwan Hwang, Dechun Feng, Bin Gao

**Affiliations:** Laboratory of Liver Diseases, National Institute on Alcohol Abuse and Alcoholism, National Institutes of Health, Bethesda, MD 20892, USA.

**Keywords:** Interleukin (IL)-22, mitochondrial protector

## Abstract

Interleukin (IL)-22 has been increasingly recognized as a promising therapeutic option for various types of diseases. This commentary summarizes the novel mechanistic aspects of IL-22 for the treatment of liver diseases including the study by Chen et al. published in the recent issue of the *Theranostics* that elucidated the novel function of IL-22 as a mitochondrial protector for the adaptive defense against liver injury.

Interleukin (IL)-22 is a member of the IL-10 family of cytokines and produced by various types of lymphocytes such as Th17, Th22, and type 3 innate lymphoid cells (ILC3s) 1. IL-22 functions by interacting with a heterodimeric receptor complex that consists of IL-10R2 and IL-22R1. Unlike the ubiquitous expression of IL-10R2, the expression of IL-22R1 is restricted mainly to epithelial cells, but not on cells of hematopoietic origin 2, which makes IL-22 unique as it is produced by immune cells but acts on non-immune cells. IL-22 has been known to play a protective role against epithelial injury in various organs such as liver, pancreas, gut, kidney, and lung by governing cell survival, regenerative, and anti-microbial capacity 3, 4.

In the liver, IL-22 plays diverse roles by acting on various types of cells such as hepatocytes, liver progenitor cells, and hepatic stellate cells 5-7. It has been reported that IL-22 protects against different types of liver injury such as T-cell mediated liver injury, chemical/drug-induced liver injury, and alcoholic liver injury, mainly by activating the signal transducers and activators of transcription 3 (STAT3) signaling pathway in hepatocytes that induces anti-apoptotic and antioxidant genes 8-13. In addition to the prevention of hepatocyte death, IL-22 also promotes liver regeneration, induction of antimicrobial genes (e.g., lipocalin 2), and suppression of lipogenic genes 9, 12, 14, 15, which may contribute to hepatoprotection, anti-infection, and metabolic regulation by IL-22. IL-22 receptor complex is also present on hepatic stellate cells, where IL-22 promotes p53/p21-dependent senescence of activated hepatic stellate cells and resolution of liver fibrosis via STAT3 activation 6. Additionally, IL-22 promotes the growth of liver progenitor cells 16, which may assist the liver to repair after chronic liver injury.

In contrast to the benefits mentioned above, IL-22 also plays a pathogenic role in a context-dependent manner 17, 18. IL-22 is elevated in patients with chronic liver diseases such as viral hepatitis and advanced liver fibrosis 5, 19. In experimental models of hepatitis B virus (HBV) infection, IL-22 has been reported to promote inflammation and fibrosis 5, 20, 21. IL-22 has been also demonstrated to promote inflammatory conditions in other organs such as skin and gut and fibrosis in the intestine 22-25. This nature of IL-22 has highlighted the therapies blocking IL-22 and its upstream inducer IL-23 for the treatment of psoriasis and inflammatory bowel disease 26. The dual nature of IL-22 is thought to be at least in part due to the presence of IL-22 binding protein (IL-22BP or IL-22Ra2), which is a soluble IL-22 receptor and an endogenous inhibitor of IL-22 27, 28. Also, it has been postulated that the function of IL-22 may be dependent on the nature of the inflammation. IL-22 may play a protective role in acute settings of inflammation by amplifying tissue repair, whereas it plays a detrimental role in chronic inflammation with a possibility to induce hyperplasia 18.

Novel perspectives on the tissue-protective role of IL-22 have been increasingly reported in the past three years. In particular, two studies published in the 2018 issues of the *Theranostics* demonstrated interesting mechanisms underlying the beneficial effect of IL-22 in the liver and the heart. First, Mo et al. identified autophagy activation as a critical mediator in IL-22's protection against acetaminophen-induced liver injury 29. Acetaminophen overdose is the leading cause of drug-induced liver injury and has been widely used to experimentally study acute liver failure. Under this condition, mitochondria-derived reactive oxygen species (ROS) leads to loss of the mitochondrial membrane potential and release of intermembrane proteins which migrate to the nucleus and stimulate nuclear DNA fragmentation. IL-22 facilitates the removal of the damaged mitochondria through AMPK activation and subsequent autophagy induction, thereby alleviating acetaminophen-induced liver injury. Also, Tang et al. reported that the action of IL-22 in hepatocytes exerts cardioprotective effect in the mouse model of myocardial infarction 30. The authors demonstrated that IL-22 treatment attenuated cardiac injury and inflammation occurring after myocardial infarction. Mechanistically, the cardioprotective effect of IL-22 was dependent on STAT3 activation and production of FGF21 in hepatocytes, which controls the expression of genes responsible for cardiomyocyte survival. This emphasizes the important role of IL-22 in the communication between the liver and the heart for an adaptive protection against cardiac injury.

Tissue protection by the inter-organ communication via IL-22 was also reported by Hendrikx et al. who have elegantly demonstrated that intestine-derived IL-22 and engineered bacteria that produce IL-22 prevent ethanol-induced liver disease 31. Under healthy conditions, gut microbiome produces indole-3-acetic acid (IAA), an aryl hydrocarbon receptor (AHR) ligand 31. IAA induces AHR-dependent production of IL-22 in gut ILC3s, which thereby promotes expression of regenerating islet-derived protein 3 gamma (REG3G) that blocks gut-to-liver bacterial translocation 31. Hendrikx et al. demonstrated that, in ethanol-fed mice, dysbiosis lowered IAA production and AHR-dependent IL-22 production, which in turn impaired REG3G-dependent maintenance of gut permeability 31, suggesting the complementary relationship between IL-22 and microbiota given that IL-22 also contributes to the homeostasis of colonic microbiota 32. When mice were supplemented with engineered bacteria that produce IL-22, ethanol-induced liver damage, inflammation, and bacterial translocation to the liver were attenuated in an REG3G-dependent manner, suggesting the possibility to treat alcoholic liver disease by IL-22 derived from an extrahepatic organ.

Mitochondria have been increasingly highlighted as the target for the protective action of IL-22 against tissue injury 33, 34. Mitochondria play a critical role in hepatic metabolism such as maintenance of lipid and glucose homeostasis 35. The metabolic regulation by hepatic mitochondria is largely dysregulated in the disease state. For example, insulin resistance alters respiration and ATP production in mitochondria which leads to aberrant ROS production 35, 36. Also, hepatic lipid metabolism such as mitochondrial fatty acid β-oxidation and *de novo* fatty acid synthesis are impaired under the pathological conditions 35, 36. In the recent issue of the *Theranostics*, Chen et al. identified IL-22 as a mitochondrial protector to control metabolic reprogramming and the maintenance of mitochondrial fitness as the novel mechanism by which IL-22 adaptively protects against liver injury 37. This previously unrecognized function of IL-22 was demonstrated by the measurement of the oxidative phosphorylation and glycolysis in damaged mouse hepatocytes. IL-22 was able to restore oxidative phosphorylation and glycolysis that were suppressed by hepatocyte damage caused by various stimuli such as CCl_4_, palmitic acid, cisplatin, and ethanol. Improvement in mitochondrial function reversed the expression of the genes involved in metabolic reprogramming such as *Hk2* and *Hif1a*, which were suppressed by hepatocyte damage. Mechanistically, the IL-22/STAT3 pathway induced lncRNA H19 which activates AMP-activated protein kinase (AMPK). Activation of AMPK mediated IL-22-dependent restoring of mitochondrial integrity and ROS reduction through AKT and mTOR activation, which is also responsible for transactivation of genes involved in glycolysis and metabolic reprogramming. Lastly, the *in vivo* relevance of IL-22-induced metabolic reprogramming was verified in mouse models of liver injury such as cisplatin treatment and high-fat diet (HFD) feeding.

While many studies have reported the protective effect of IL-22 against various types of liver injury, acute-on-chronic liver failure (ACLF) and nonalcoholic steatohepatitis (NASH) have been relatively unexplored in terms of the therapeutic potential of IL-22, partially due to the lack of appropriate animal models to study these diseases. Recently, we have developed novel models of ACLF and NASH in mice and assessed the therapeutic benefit of IL-22 in these models. For the new mouse ACLF model, mice were challenged by combination of chronic CCl_4_ administration with acute CCl_4_ challenge and bacterial infection 38. In this model, liver regeneration was blocked through the inhibition of the pro-regenerative IL-6/STAT3 pathway but activation of the anti-regenerative interferon-γ/STAT1 pathway, which was similarly observed in ACLF patients. IL-22 treatment ameliorated ACLF by activating STAT3 pathway and inducing anti-bacterial genes via the STAT3/BCL2 pathway, which supports the feasibility of the development of IL-22 therapy for the treatment of ACLF.

We also established a new HFD*^+Cxcl1^*-induced NASH model based on the clinical observation that neutrophil infiltration is markedly increased, and neutrophil-recruiting chemokines (e.g., C-X-C motif ligand 1, CXCL1) are upregulated in the liver of NASH patients compared to fatty liver 39. Hepatic overexpression of *Cxcl1* in 3-month HFD-fed mice significantly increased neutrophil infiltration, which promoted neutrophil-derived oxidative stress, liver injury, inflammation, and fibrosis compared to mice fed an HFD only 40. Interestingly, activation of p38 mitogen-activated protein kinase protects against HFD-induced steatosis but exacerbates HFD*^+Cxcl1^*-induced NASH in this HFD*^+Cxcl1^* model 41. Administration of IL-22 ameliorated the HFD*^+Cxcl1^*-induced NASH through activation of metallothionein-1 and -2, which are potent antioxidant enzymes under the transcriptional regulation of the IL-22/STAT3 pathway 40. This finding from our study added NASH to the list of diseases potentially amenable to IL-22 therapy.

The half-life of IL-22 is less than 2 hours, which has hindered its development as a therapeutic option 42. For this reason, companies such as Generon Corporation and Genentech have developed engineered IL-22 molecules to a dimer to extend its half-life for clinical application. The majority of the recent studies mentioned above utilized IL-22Fc, a recombinant fusion protein consisting of two human IL-22 molecules linked to an immunoglobulin constant region (IgG2-Fc) with a prolonged half-life (39 h-206 h) 42. In two phase 1 clinical trials, IL-22Fc showed safety and well-tolerance with favorable pharmacokinetic properties 42, 43. Also, in a phase 2a open-label study for alcoholic hepatitis, IL-22Fc ameliorated moderate and severe alcoholic hepatitis with an improvement in Lille and model for end-stage liver disease (MELD) scores accompanied by a decrease in inflammatory markers and increase in hepatic regeneration markers 44. These promising results from the clinical studies corroborate the results from *in vitro* and *in vivo* studies that advocate the feasibility of IL-22 therapy for the treatment of liver diseases.

Despite continuing efforts to discover the pathogenic mechanisms and therapeutic targets, few medications have been approved for the treatment of liver diseases. As discussed in this article, accumulating literature has documented the basic biology of IL-22 and the molecular mechanisms by which IL-22 functions, and recent advances in the study of IL-22 have established its therapeutic efficacy in experimental models of liver diseases. Moreover, the recent development of engineered recombinant IL-22 protein has overcome the inherent difficulty of the therapeutic development of IL-22 and accelerated the clinically relevant discoveries supporting the introduction of IL-22 therapy. With promising results from phase I and phase II clinical trials, IL-22 has been now highlighted as a safe option for the treatment of multiple diseases including alcoholic hepatitis. Although concerns have been raised regarding the oncogenic ability of IL-22, animal studies have revealed that administration of IL-22 alone is hardly able to develop liver tumors spontaneously without preexisting oncogenic cues at least in short-term studies 12. Overall, IL-22 has a great potential to be developed as the therapeutic agent for a multitude of liver diseases.

## Figures and Tables

**Figure 1:**
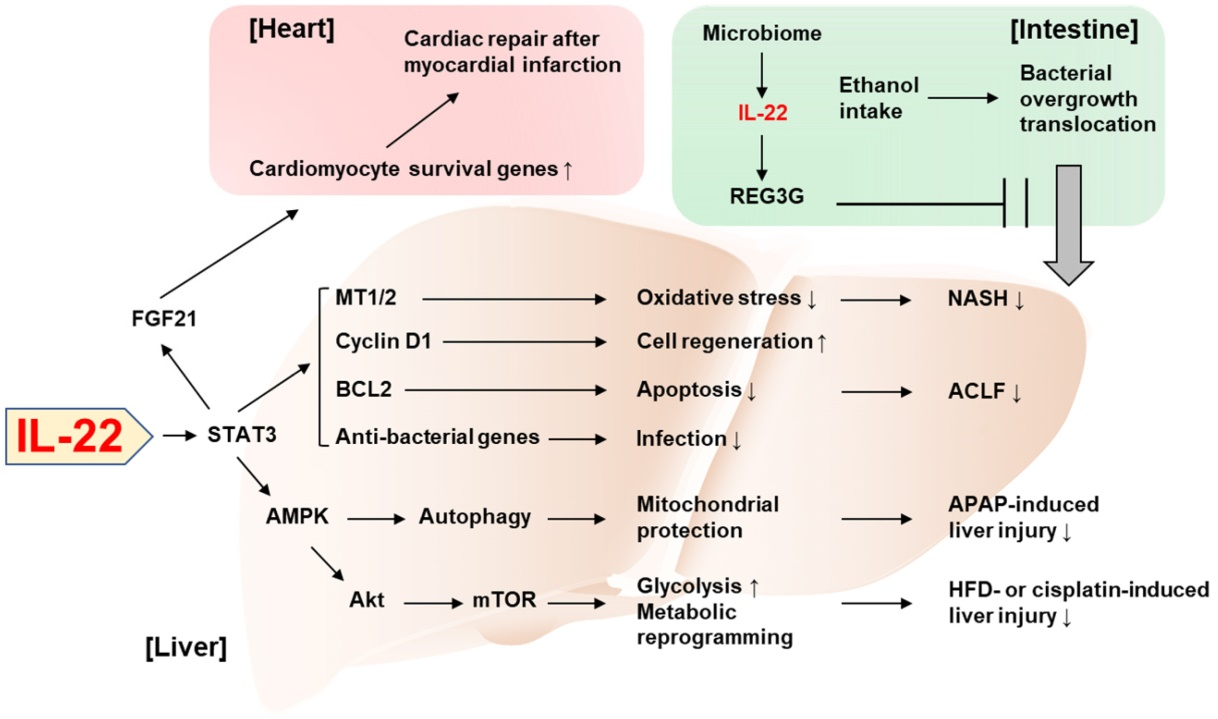
** Recent discoveries on the protective role of IL-22 against tissue injury.** IL-22 ameliorates NASH via STAT3-dependent induction of antioxidant enzymes, MT1/2. IL-22/STAT3-dependent induction of cyclin D1, BCL2, and anti-bacterial genes as well as MT1/2 contributes to the amelioration of ACLF. The IL-22/STAT3/AMPK pathway promotes autophagy-dependent mitochondrial protection, which in turn protects against APAP-induced liver injury. AMPK/AKT/mTOR signaling is also activated by IL-22, which induces genes involved in glycolysis and metabolic reprogramming, thereby suppressing the liver injury caused by HFD feeding or cisplatin treatment. The IL-22/STAT3 pathway upregulates FGF21 in hepatocytes, which in turn upregulates cardiomyocyte survival genes and repairs myocardial infarction-derived cardiac injury. IL-22 produced by intestinal innate lymphoid cells upregulates REG3G that inhibits ethanol-induced bacterial growth and translocation to the liver, thereby protecting against alcohol-induced liver injury. ACLF: acute-on-chronic liver failure; AMPK: AMP-activated protein kinase; APAP: acetaminophen; BCL2: B cell lymphoma/leukemia-2; FGF21: fibroblast growth factor 21; HFD: high-fat diet; MT: metallothionein; mTOR: mammalian target of rapamycin; NASH: nonalcoholic steatohepatitis; REG3G: regenerating islet-derived protein 3 gamma; STAT3: signal transducers and activators of transcription 3.

## References

[1] Dudakov JA, Hanash AM, van den Brink MR (2015). Interleukin-22: immunobiology and pathology. Annu Rev Immunol.

[2] Rutz S, Eidenschenk C, Ouyang W (2013). IL-22, not simply a Th17 cytokine. Immunol Rev.

[3] Sabat R, Ouyang W, Wolk K (2014). Therapeutic opportunities of the IL-22-IL-22R1 system. Nat Rev Drug Discov.

[4] Cao Q, Gao X, Lin Y, Yue C, Wang Y, Quan F (2019). Thymopentin ameliorates dextran sulfate sodium-induced colitis by triggering the production of IL-22 in both innate and adaptive lymphocytes. Theranostics.

[5] Feng D, Kong X, Weng H, Park O, Wang H, Dooley S (2012). Interleukin-22 promotes proliferation of liver stem/progenitor cells in mice and patients with chronic hepatitis B virus infection. Gastroenterology.

[6] Kong X, Feng D, Wang H, Hong F, Bertola A, Wang FS (2012). Interleukin-22 induces hepatic stellate cell senescence and restricts liver fibrosis in mice. Hepatology.

[7] Radaeva S, Sun R, Pan HN, Hong F, Gao B (2004). Interleukin 22 (IL-22) plays a protective role in T cell-mediated murine hepatitis: IL-22 is a survival factor for hepatocytes via STAT3 activation. Hepatology.

[8] Kong X, Feng D, Mathews S, Gao B (2013). Hepatoprotective and anti-fibrotic functions of interleukin-22: therapeutic potential for the treatment of alcoholic liver disease. J Gastroenterol Hepatol.

[9] Ki SH, Park O, Zheng M, Morales-Ibanez O, Kolls JK, Bataller R (2010). Interleukin-22 treatment ameliorates alcoholic liver injury in a murine model of chronic-binge ethanol feeding: role of signal transducer and activator of transcription 3. Hepatology.

[10] Lai R, Xiang X, Mo R, Bao R, Wang P, Guo S (2015). Protective effect of Th22 cells and intrahepatic IL-22 in drug induced hepatocellular injury. J Hepatol.

[11] Pan H, Hong F, Radaeva S, Gao B (2004). Hydrodynamic gene delivery of interleukin-22 protects the mouse liver from concanavalin A-, carbon tetrachloride-, and Fas ligand-induced injury via activation of STAT3. Cell Mol Immunol.

[12] Park O, Wang H, Weng H, Feigenbaum L, Li H, Yin S (2011). In vivo consequences of liver-specific interleukin-22 expression in mice: Implications for human liver disease progression. Hepatology.

[13] Scheiermann P, Bachmann M, Goren I, Zwissler B, Pfeilschifter J, Muhl H (2013). Application of interleukin-22 mediates protection in experimental acetaminophen-induced acute liver injury. Am J Pathol.

[14] Zheng Y, Valdez PA, Danilenko DM, Hu Y, Sa SM, Gong Q (2008). Interleukin-22 mediates early host defense against attaching and effacing bacterial pathogens. Nat Med.

[15] Yang L, Zhang Y, Wang L, Fan F, Zhu L, Li Z (2010). Amelioration of high fat diet induced liver lipogenesis and hepatic steatosis by interleukin-22. J Hepatol.

[16] Brand S, Dambacher J, Beigel F, Zitzmann K, Heeg MH, Weiss TS (2007). IL-22-mediated liver cell regeneration is abrogated by SOCS-1/3 overexpression in vitro. Am J Physiol Gastrointest Liver Physiol.

[17] Brockmann L, Giannou AD, Gagliani N, Huber S (2017). Regulation of T(H)17 cells and associated cytokines in wound healing, tissue regeneration, and carcinogenesis. Int J Mol Sci.

[18] Zenewicz LA (2018). IL-22: There Is a Gap in Our Knowledge. Immunohorizons.

[19] Fabre T, Molina MF, Soucy G, Goulet JP, Willems B, Villeneuve JP (2018). Type 3 cytokines IL-17A and IL-22 drive TGF-β-dependent liver fibrosis. Sci Immunol.

[20] Zhang Y, Cobleigh MA, Lian JQ, Huang CX, Booth CJ, Bai XF (2011). A proinflammatory role for interleukin-22 in the immune response to hepatitis B virus. Gastroenterology.

[21] Zhao J, Zhang Z, Luan Y, Zou Z, Sun Y, Li Y (2014). Pathological functions of interleukin-22 in chronic liver inflammation and fibrosis with hepatitis B virus infection by promoting T helper 17 cell recruitment. Hepatology.

[22] Zhang Y, Xia Q, Li Y, He Z, Li Z, Guo T (2019). CD44 assists the topical anti-psoriatic efficacy of curcumin-loaded hyaluronan-modified ethosomes: A new strategy for clustering drug in inflammatory skin. Theranostics.

[23] Mathur R, Alam MM, Zhao XF, Liao Y, Shen J, Morgan S (2019). Induction of autophagy in Cx3cr1(+) mononuclear cells limits IL-23/IL-22 axis-mediated intestinal fibrosis. Mucosal Immunol.

[24] Kamanaka M, Huber S, Zenewicz LA, Gagliani N, Rathinam C, O'Connor W Jr (2011). Memory/effector (CD45RB(lo)) CD4 T cells are controlled directly by IL-10 and cause IL-22-dependent intestinal pathology. J Exp Med.

[25] Eken A, Singh AK, Treuting PM, Oukka M (2014). IL-23R+ innate lymphoid cells induce colitis via interleukin-22-dependent mechanism. Mucosal Immunol.

[26] Moschen AR, Tilg H, Raine T (2019). IL-12, IL-23 and IL-17 in IBD: immunobiology and therapeutic targeting. Nat Rev Gastroenterol Hepatol.

[27] Kleinschmidt D, Giannou AD, McGee HM, Kempski J, Steglich B, Huber FJ (2017). A protective function of IL-22BP in ischemia reperfusion and acetaminophen-induced liver injury. J Immunol.

[28] Pelczar P, Witkowski M, Perez LG, Kempski J, Hammel AG, Brockmann L (2016). A pathogenic role for T cell-derived IL-22BP in inflammatory bowel disease. Science.

[29] Mo R, Lai R, Lu J, Zhuang Y, Zhou T, Jiang S (2018). Enhanced autophagy contributes to protective effects of IL-22 against acetaminophen-induced liver injury. Theranostics.

[30] Tang TT, Li YY, Li JJ, Wang K, Han Y, Dong WY (2018). Liver-heart crosstalk controls IL-22 activity in cardiac protection after myocardial infarction. Theranostics.

[31] Hendrikx T, Duan Y, Wang Y, Oh JH, Alexander LM, Huang W (2019). Bacteria engineered to produce IL-22 in intestine induce expression of REG3G to reduce ethanol-induced liver disease in mice. Gut.

[32] Zenewicz LA, Yin X, Wang G, Elinav E, Hao L, Zhao L (2013). IL-22 deficiency alters colonic microbiota to be transmissible and colitogenic. J Immunol.

[33] Chen W, Zhang X, Fan J, Zai W, Luan J, Li Y (2017). Tethering interleukin-22 to apolipoprotein A-I ameliorates mice from acetaminophen-induced liver injury. Theranostics.

[34] Shen Y, Jin X, Chen W, Gao C, Bian Q, Fan J Interleukin-22 ameliorated acetaminophen-induced kidney injury by inhibiting mitochondrial dysfunction and inflammatory responses. Appl Microbiol Biotechnol, in press. doi: 10.1007/s00253-020-10638-4.

[35] Degli Esposti D, Hamelin J, Bosselut N, Saffroy R, Sebagh M, Pommier A (2012). Mitochondrial roles and cytoprotection in chronic liver injury. Biochem Res Int.

[36] Auger C, Alhasawi A, Contavadoo M, Appanna VD (2015). Dysfunctional mitochondrial bioenergetics and the pathogenesis of hepatic disorders. Front Cell Dev Biol.

[37] Chen W, Zai W, Fan J, Zhang X, Zeng X, Luan J (2020). Interleukin-22 drives a metabolic adaptive reprogramming to maintain mitochondrial fitness and treat liver injury. Theranostics.

[38] Xiang X, Feng D, Hwang S, Ren T, Wang X, Trojnar E (2020). Interleukin-22 ameliorates acute-on-chronic liver failure by reprogramming impaired regeneration pathways in mice. J Hepatol.

[39] Bertola A, Bonnafous S, Anty R, Patouraux S, Saint-Paul MC, Iannelli A (2010). Hepatic expression patterns of inflammatory and immune response genes associated with obesity and NASH in morbidly obese patients. PLoS One.

[40] Hwang S, He Y, Xiang X, Seo W, Kim SJ, Ma J Interleukin-22 ameliorates neutrophil-driven nonalcoholic steatohepatitis through multiple targets. Hepatology, in press.

[41] Hwang S, Wang X, Rodrigues RM, He Y, Seo W, Ma J Protective and detrimental roles of p38a MAPK in different stages of nonalcoholic fatty liver disease. Hepatology, in press.

[42] Tang KY, Lickliter J, Huang ZH, Xian ZS, Chen HY, Huang C (2019). Safety, pharmacokinetics, and biomarkers of F-652, a recombinant human interleukin-22 dimer, in healthy subjects. Cell Mol Immunol.

[43] Rothenberg ME, Wang Y, Lekkerkerker A, Danilenko DM, Maciuca R, Erickson R (2019). Randomized phase I healthy volunteer study of UTTR1147A (IL-22Fc): A potential therapy for epithelial injury. Clin Pharmacol Ther.

[44] Arab JP, Sehrawat TS, Simonetto DA, Verma VK, Feng D, Tang T An open label, dose escalation study to assess the safety and efficacy of IL-22 agonist F-652 in patients with alcoholic hepatitis. Hepatology, in press.

